# Fungal communities and their association with nitrogen-fixing bacteria affect early decomposition of Norway spruce deadwood

**DOI:** 10.1038/s41598-020-64808-5

**Published:** 2020-05-15

**Authors:** María Gómez-Brandón, Maraike Probst, José A. Siles, Ursula Peintner, Tommaso Bardelli, Markus Egli, Heribert Insam, Judith Ascher-Jenull

**Affiliations:** 1 Grupo de Ecoloxía Animal (GEA), Universidade de Vigo, E-36310 Vigo, Spain; 2 Department of Microbiology, University of Innsbruck, Technikerstraβe 25, A-6020 Innsbruck, Austria; 3 Department of Plant and Microbial Biology, University of California at Berkeley, Berkeley, CA 94720 USA; 4 Dipartimento di Scienze e Tecnologie Agrarie, Alimentari, Ambientali e Forestali (DAGRI), University of Florence, Piazzale delle Cascine 18, I-50144 Florence, Italy; 5 Council for Research and Experimentation in Agriculture (CREA-ZA), Via A. Lombardo 11, I-26900 Lodi, Italy; 6 Department of Geography, University of Zürich, Winterthurerstraße 190, CH-8057 Zürich, Switzerland

**Keywords:** Ecology, Microbial ecology

## Abstract

Deadwood decomposition is relevant in nature and wood inhabiting fungi (WIF) are its main decomposers. However, climate influence on WIF community and their interactions with bacteria are poorly understood. Therefore, we set up an *in-field* mesocosm experiment in the Italian Alps and monitored the effect of slope exposure (north- vs. south-facing slope) on the decomposition of *Picea abies* wood blocks and their microbiome over two years. Unlike fungal richness and diversity, we observed compositional and functional differences in the WIF communities as a function of exposure. Wood-degrading operational taxonomic units (OTUs) such as *Mycena*, and mycorrhizal and endophytic OTUs were characteristic of the south-facing slope. On the north-facing one, Mucoromycota, primarily *Mucor*, were abundant and mixotrophic basidiomycetes with limited lignin-degrading capacities had a higher prevalence compared to the southern slope. The colder, more humid conditions and prolonged snow-coverage at north exposure likely influenced the development of the wood-degrading microbial communities. Networks between WIF and N_2_-fixing bacteria were composed of higher numbers of interacting microbial units and showed denser connections at the south-facing slope. The association of WIF to N_2_-fixing *Burkholderiales* and *Rhizobiales* could have provided additional competitive advantages, especially for early wood colonization.

## Introduction

Deadwood is an important temporary carbon pool in forest ecosystems^1^. Its composition comprises simple sugars and organic acids along with complex biopolymers, primarily cellulose and lignin, which makes deadwood a resource of difficult access and decomposition for most organisms^2^. Saprotrophic fungi from the phylum Basidiomycota are considered the primary wood decomposers^3^; among them, white-rot fungi play a pivotal role in deadwood decomposition due to their ability for lignin degradation with the aid of a plethora of extracellular lignocellulolytic enzymes^4^. Brown-rot basidiomycetes also play an important role in the decomposition of deadwood, as they are capable of oxidizing cellulose via a non-enzymatic mechanism^5^. In addition to Basidiomycota, some Ascomycetes are capable of decomposing celluloses and hemicelluloses of the secondary cell wall causing a spongy texture at the wood surface (soft-rot)^2^. Moreover, the presence of Ascomycota may influence the wood decomposition rate by interacting or competing with Basidiomycota at least in the early stage of decomposition^3^. Other functional groups such as ectomycorrhizal, lichenized, mycoparasitic and plant pathogenic fungi can also be associated with deadwood by mediating the interactions between plants, arthropods and fungal saprotrophs^6^ and in turn, affect forest ecosystem functions and services including nutrient cycling and global carbon dynamics.

Field surveys on wood-inhabiting fungi (WIF) have traditionally been confined to fruiting body surveys or mycelia isolations^3,7^; however, these culture-dependent approaches do not provide total community evaluation^3,8^. Recently, high-throughput sequencing has offered a more comprehensive picture of the drivers shaping the deadwood fungal biota across different forest biomes^3,6,9^. At one end, environmental factors which are known to affect WIF composition and diversity include the wood physico-chemical properties such as density, size, moisture, pH and nutrient content^3,10,11^. The decomposition stage of wood^12^ and the soil type^4^ are also important factors. At the other end, biotic drivers also affect WIF richness and diversity. These are related to the host tree species^3,13^, the fungal community assembly^14,15^, the interactions between WIF and saproxylic insects^16,17^, and the interactions between fungal and bacterial deadwood colonizers^18–21^.

There is ever increasing evidence about the occurrence of bacterial-fungal interactions within the deadwood environment, ranging from mutualism to antagonism, as reviewed by Johnston *et al*.^20^. Nonetheless, the role of bacteria in decomposing wood has been underestimated despite evident mutualistic relationships between N_2_-fixing bacteria and WIF communities^18,22,23^. Nitrogen (N) availability in deadwood is highly restricted, suggesting that WIF may take advantage from associations with N_2_-fixing bacteria to meet their N requirements for vegetative growth and propagation. In line with this, Hoppe *et al*.^18^ reported for the first time a positive correlation between the number of fruiting bodies and the diversity of the N-related *nif*H gene in *Picea abies* and *Fagus sylvatica* logs. Moreover, the wood decomposition rates of these two temperate tree species positively correlated with the richness of fruiting bodies and N_2_-fixing bacteria^23^.

Topographic features such as slope exposure may have important consequences on deadwood decomposition process^24^ and on the underlying soil microbial communities as recently shown in a 2-year mesocosm monitoring of *P. abies* wood decomposition performed at the same experimental site in the Italian Alps^25–28^. A faster deadwood decomposition rate at the south-facing slope with respect to the north-facing one was related to a higher bacterial richness and a higher number of detected specialist operational taxonomic units (OTUs)^28^. However, it is still unclear whether and how exposure affects the interactions between fungal and bacterial deadwood colonizers.

At an elevation of about 2,000 m above sea level (a.s.l., close to the tree line) and over a decomposition period of two years (104 weeks), we used *in-field* mesocosms to evaluate the microbial-mediated decomposition process of deadwood. Internal transcribed spacer (ITS) amplicon sequencing was applied to analyse the fungal communities in *P. abies* experimental wood blocks and their underlying soil. The main aims were to (1) evaluate the impact of exposure (north- *vs*. south-facing slope) on the fungal taxonomic and guild composition; and (2) identify potential fungal key taxa in *P. abies* wood decomposition. Together with the bacterial dataset from the very same mesocosm experiment^28^, we further aimed to (3) determine the interactions between fungi and N_2_-fixing bacteria as a function of slope exposure and progressing wood decomposition.

Along with an increase in the wood’s surface area and nutrient variety as decomposition progressed, we expected a higher number of available ecological niches for microbial colonization. Consequently, we hypothesized that fungal richness and diversity in the *P. abies* wood blocks would increase over the 2-year observational period, reaching higher values at the south- rather than at the north-facing slope considering the faster deadwood decomposition rate at the southern exposure^24^. In light of this, we also expected that a denser network would characterize the south-facing slope with greater connectivity between fungal and N_2_-fixing bacterial OTUs compared to the north-facing one.

## Results and discussion

### Influence of slope exposure on decay rates of the *P. abies* wood blocks

**Figure 1:**
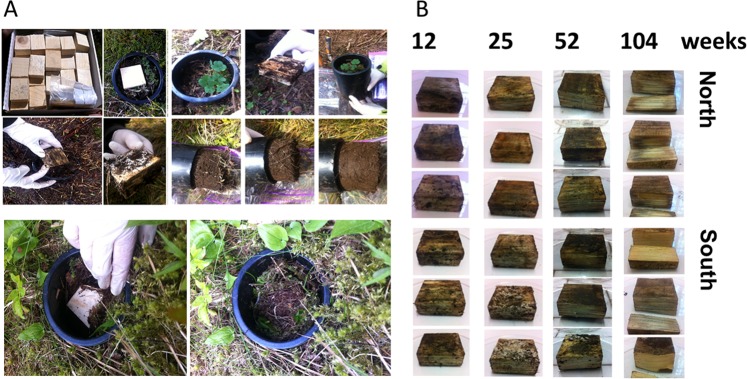
Overview of the (**A**) destructive sampling procedure of the *P. abies* experimental wood blocks and the underlying soil (0–5 cm); and (**B**) wood blocks at the different time points (12, 25, 52 and 104 weeks) over the 2-year observational period at the north- and the south-facing site.

Two years after the placement of the wood blocks onto the soil mesocosms (Figs. 1, S1), the decomposition of the *P. abies* wood blocks was still in its early stages. This is in line with the lack of differences in mass loss within the 2-year period (Table 1). However, a lower cellulose content was reported after 104 weeks compared to the earlier time points, and such differences over time were more pronounced at the south-facing slope (Table 1). The lowest moisture content and pH values were observed at the end of the trial (Table 1). While pH was not influenced by slope exposure (Table 1), moisture was lower in the south- than in the north-exposed wood blocks (Table 1). In a representative year (2013/14), snow covered the experimental mesocosms at both slopes as shown in the temperature profile (Fig. S2). However, the mesocosms at the south-facing slope were free of snow at the end of April, while the snow cover remained at the north-facing slope until the end of May. The prolonged snow coverage and consequently the longer period at zero degrees might have affected the wood decomposition rates and the associated microbial activity at the two slope exposures.

**Table 1:** Physico-chemical properties of the *Picea abies* experimental wood blocks collected in June 2013 (t0), and from the mesocoms in August 2013 (t1; 12 weeks), in October 2013 (t2; 25 weeks), in July 2014 (t3; 52 weeks), and in July 2015 (t4; 104 weeks) at the north and the south facing slopes.

Sites	Sampling time	Moisture (%)	pH	Cellulose (%)	Mass (g)
North exposure	t0	8.24 ± 2.01	5.54 ± 0.11	42.67 ± 5.28	22.5 ± 3.36
	t1	54.92 ± 6.42	5.50 ± 0.07	45.25 ± 2.77	22.4 ± 6.17
	t2	60.99 ± 1.85	5.42 ± 0.22	46.21 ± 3.05	21.6 ± 1.82
	t3	61.11 ± 2.25	5.74 ± 0.30	45.91 ± 2.23	18.0 ± 1.99
	t4	44.98 ± 9.08	5.41 ± 0.23	37.73 ± 3.30	22.4 ± 1.22
South exposure	t1	52.64 ± 6.40	5.71 ± 0.05	45.44 ± 2.12	19.1 ± 3.44
	t2	47.26 ± 5.86	5.41 ± 0.30	46.29 ± 3.21	20.2 ± 4.16
	t3	58.98 ± 5.42	5.44 ± 0.17	45.53 ± 2.90	20.5 ± 1.72
	t4	12.82 ± 2.74	4.85 ± 0.24	31.07 ± 6.71	17.1 ± 3.96
Linear model		time = −6.87	time = −0.095	time = −3.34	time = n.s.
		p_time_ = 0.0053	p_time_ = 0.0289	p_time_ = 0.0012	exposure = n.s.
		exposure_N_ = 64.4	R^2^ = 0.144	exposure_N_ = 9.44	
		p_exposureN_ = 8e-7	exposure = n.s.	p_exposureN_ = 0.024	
		exposure_S_ = 51.9		exposure_S_ = 7.75	
		p_exposureS_ = 2e-5		p_exposureS_ = 0.059	
		R^2^ = 0.623		R^2^ = 0.306	

Values are means ± standard deviation (n = 3). In order to assess significance, a linear model was fitted using the slope exposure and the time as fixed factors. Backwards selection of factors was used for model selection.

n.s: no significant.

### WIF richness and diversity were neither influenced by slope exposure nor time

Illumina analysis yielded a total of > 5.3 million reads (wood and soil), from which 93,546 ± 29,127 quality filtered sequences were obtained per sample after quality trimming and removal of non-target and chimeric sequences. These reads were clustered into 5,009 fungal OTUs at 97% sequence identity. The removal of rare OTUs (less than 5 reads per sample and observed in less than 3 samples) did not cause any effect on the fungal community composition (Procrustes m^2^ = 0.000222). The remaining 3,555 abundant fungal OTUs were retained for further analysis. The sample-based rarefaction curves indicated saturation of fungal diversity at the analysed sequencing depth for wood and soil samples (Fig. S3).

**Figure 2:**
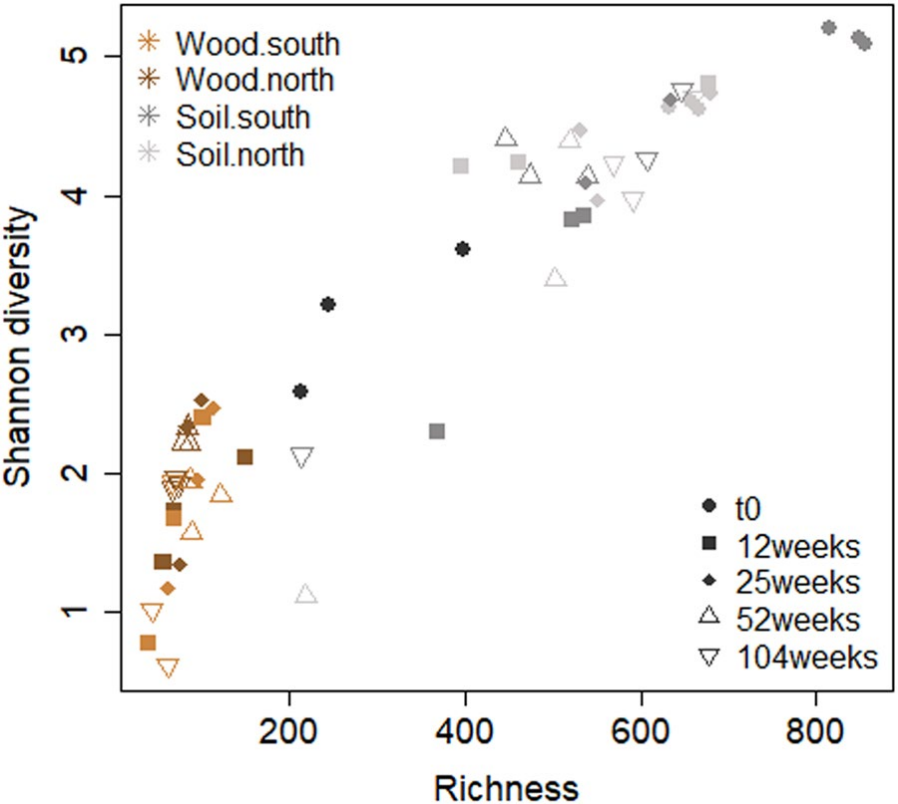
Relationship between fungal richness and Shannon diversity of the *P. abies* experimental wood blocks and the underlying soil samples (0–5 cm) at the different time points (12, 25, 52 and 104 weeks) over the 2-year observational period at the north- and the south-facing site.

Soil samples were more diverse and had a higher OTU richness compared to the *P. abies* wood blocks (Fig. 2). The highest WIF richness and Shannon diversity was reported for t0 (Fig. 2). Both measures, richness and Shannon diversity, were independent of slope exposure (p_richness_ = 0.84, p_Shannon diversity_ = 0.11) and time (p_richness_ > 0.24, p_Shannon diversity_ = 0.24). However, under the same experimental conditions, the south-exposed *P. abies* wood blocks were characterized by higher bacterial richness and Shannon diversity with progressing decay^28^. This might be related to the faster rates of turnover in bacterial communities^29^, which make them more sensitive and consequently react more quickly than fungi to the physical and chemical changes as woody material decomposes. Indeed, neither WIF richness nor diversity were significantly influenced by pH (p_richness_ = 0.42, p_Shannon diversity_ = 0.88), moisture (p_richness_ = 0.23, p_Shannon diversity_ = 0.25) and cellulose content (p_richness_ = 0.90, p_Shannon diversity_ = 0.86) in the two study sites. This is in line with litter transplantation experiments^29^, in which fungi were found to be more resistant to change and less reflective of their new environment than bacteria.

**Figure 3:**
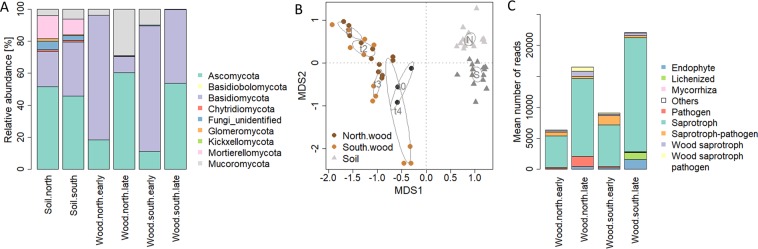
Taxonomic and functional composition of the *P. abies* experimental *w*ood blocks and the underlying soil samples. (**A**) Distribution of taxa across wood blocks and soil samples. Operational taxonomic units (OTUs) detected by Illumina Miseq sequencing of the fungal ITS2 region were summarized based on their phylum annotation. (**B**) Non-metric multidimensional scaling (NMDS) based on Bray Curtis dissimilarities between the OTU compositions of the samples. Lowest stress was 0.139. The iteration converged after 20 tries. (**C**) The guild annotation of OTUs detected in all samples was predicted by their taxonomic annotation using FUNGuild^32^. The functional composition of the samples was illustrated as the abundance of OTUs assigned to the guilds. FUNGuild annotations were manually summarized into fewer categories in order to simplify visualization and data accession (Table S1).

The composition of WIF communities significantly differed from the respective underlying soil fungal communities (R^2^ = 0.24, p_Adonis_ = 0.001; Fig. 3B). While the main part of the variance among the soil samples was attributed to the slope exposure (R^2^ = 0.24, p = 0.001; Fig. 3B), the *P. abies* wood blocks’ fungal composition mainly followed a time trajectory at both slope exposures (R^2^ = 0.33, p_Adonis_ = 0.001; Fig. 3B). Further supporting the dependency between WIF community and deadwood decomposition progression, the experimental wood blocks’ fungal communities at the two study sites diverged over time, despite coming from the same tree (interaction effect of time and exposure, R^2^ = 0.11, p_Adonis_ = 0.002) (Fig. 3B). A clearer differentiation was found between the north- and the south-exposed wood blocks after 25 (t2) and 52 (t3) weeks (p_time_ = 0.002, p_exposure_ = 0.161). After 52 (t3) and 104 (t4) weeks, differences between WIF communities were also significant and accounted for over 20% of the variance (p_time_ = p_exposure_ = p_time*exposure_ = 0.001; Fig. 3B). As occurred with WIF diversity and richness, neither cellulose nor moisture contents were significantly related to WIF composition based on the Adonis analysis (p_cellulose_ = 0.148, p_moisture_ = 0.062). However, pH appeared to be correlated with changes in WIF community composition accounting for 14% of the variance (p_Adonis_ = 0.002). Accordingly, Purahong *et al*.^6^ found wood pH as the only factor that consistently corresponded to WIF community composition in broadleaved and coniferous tree species after a 3-year decomposition time.

### WIF composition and indicator species differed in their taxonomy and ecologic function between the north- and the south-facing slope

In order to understand how WIF contributed to the differential deadwood decomposition, we compared the distribution of WIF taxa and their annotated functions between the north- and the south-facing site. Overall, the fungal community of the *P. abies* wood blocks mainly consisted of Basidiomycota and Ascomycota (36% and 53% of the sequences, respectively; Fig. 3A). Supporting Purahong *et al*.^13^, our *P. abies* blocks were characterized by lower numbers of Basidiomycota OTUs (mean_Ascomycota_ = 63, mean_Basidiomycota_ = 30, pWilcox = 0.003). This is consistent with the findings by Longa *et al*.^7^, who isolated more Asco- than Basidiomycota from the experimental wood blocks analysed in the present study. In our study, the most abundant Basidiomycota OTUs belonged to *Guehomyces* and *Mycena* (54% and 12% of Basidiomycota reads, respectively). In the case of Ascomycetes, *Pleosporales* and *Helotiales* were the most abundant, accounting for 35% and 21% of all Ascomycota reads, respectively. At the earlier time points (12 and 25 weeks), the relative abundance of Basidiomycota reads exceeded those from Ascomycota at both study sites (Fig. 3A); while the opposite trend was observed after 52 and 104 weeks (Fig. 3A). In line with their abundance, the richness of Basidiomycota also decreased over time, and mainly at the north-facing slope (Spearman’s rho_North_ = −0.85, p = 5e-4; Spearman’s rho_South_ = −0.76, p = 0.004). However, the OTU richness of Ascomycota did not correlate with time (Spearman’s rho = 0.2, p = 0.33), suggesting that higher species richness is not necessarily related to faster wood degradation rates as pointed out by Rinne-Garmston *et al*.^30^.

Mucoromycota also accounted for a significant percentage of reads in the wood blocks (11%) and were almost exclusively annotated to the genus *Mucor* (99%). This phylum was detected on the north- and the south-exposed wood blocks at the earlier time points (Fig. 3A). However, after 52 and 104 weeks, their presence was almost negligible on the south-exposed wood blocks while it accounted for over 20% of all reads in those collected at the north-facing site (Fig. 3A). This is in agreement with the preference of *Mucor* spp. for colder environments^31^.

From a functional perspective, a total of 1,333 OTUs (956 OTUs from the filtered OTU table, that is 27% of all OTUs detected) were assigned to an ecological role using FUNGuild^32^. Saprotrophic fungi (general, wood and potentially pathogenic saprotrophs) accounted for 82% of reads and thus, for the majority of the functionally annotated OTUs on both north- and south-exposed wood blocks regardless of the time point (Figs. 3C; S4A,B; Table S1). However, at the later sampling points (52 and 104 weeks), WIF functional composition varied with exposure (Fig. S4C), and the south-exposed wood blocks were characterized by a higher abundance and frequency of OTUs annotated as lichenized (mean_north.late_ = absent, mean_south.late_ = 1.1%), mycorrhizal (mean_north.late_ = 0.03%, mean_south.late_ = 0.2%) and endophytic (mean_north.late_ = 3.8%; mean_south.late_ = 5.3%), and by a lower abundance of OTUs classified as potentially pathogenic (mean_north.late_ = 9.3%; mean_south.late_ = 7.8%; Fig. 3C, Table S1).

Having found differences in both WIF taxonomic (at phylum level) and functional composition between the north- and the south-exposed wood blocks, we sought to predict the indicator species that might act as key players during the 2-year period by using the linear discriminant analysis (LDA) effect size (LEfSe) tool. Considering only OTUs that were observed three times between 12 and 104 weeks, *primary colonising* OTUs were defined as OTUs that were present at t1 (12 weeks) and absent at t4 (104 weeks); and *secondary colonising* OTUs as those absent at t1 (12 weeks) and present at t4 (104 weeks). Indicator species and *primary/secondary colonizers* often coincided (Table S2). Among the early wood colonizers (Table S2), we mainly found basidiomycete yeasts from the classes *Tremellomycetes* (*Cryptococcus* sp., *Cystofilobasidium* sp.) and *Microbotryomycetes* (*Rhodosporidium kratochvilovae*). Another key player at the earlier stages was *Guehomyces pullulans* (*= Aureobasidium pullulans)*, which is an ubiquitous yeast-like fungus that can colonize weathered wood, utilising products of biotic and abiotic lignocellulose degradation^33^. *G. pullulans* is very efficient in conquering the habitat in the very early stages of wood decomposition. As decomposition proceeds, its biomass can be used by other species providing a valuable nutrient source^33^. However, it should be noted that our experimental design focused on the decay dynamics of relatively small and equally sized wood blocks, and key attributes determining the WIF community composition include also the wood diameter^34^. Altogether, these circumstances could have favoured the presence and higher contribution of basidiomycete yeasts as important players during the early stages of decomposition of our *P. abies* wood blocks within the 2-year period.

Fungal indicator species of the later stages of wood decomposition (52 and 104 weeks), as detected by LefSe, clearly differed as a function of slope exposure. Indicator species of the north-exposed wood blocks were often Mucoromycota, as represented by five *Mucor* species (Table S2). Although Mucoromycota are not considered typical deadwood composers, recent research suggests that they are involved in wood decomposition via facilitating the breakdown of complex sugars^35^. Another interesting indicator species for the later stages of decomposition at the northern slope was the ascomycete *Trichoderma viride* (Table S2). *Trichoderma* species are poor lignin- but effective cellulose decomposers and benefit from the delignification process performed by white-rot fungi. They also act as antagonists to other fungal species, including mycoparasitic interaction in the wood using other mycelia as resources^36^.

At the south-facing slope, indicator species for the later stages included key players with different functions, such as the wood decomposing species *Heterochaetella brachyspora*, *Sarea difformis* and *Mycena flavoalba*^35,37^ (Tables S1 and S2). In addition, the mycorrhizal fungi *Meliniomyces bicolor* and *Cladophialophora* appeared as indicator species in the later south-exposed wood blocks (Tables S1 and S2), likely due to the earlier snowmelt and longer vegetation periods at this slope exposure. Accordingly, the underlying soil was also characterized by a higher abundance of the ericoid mycorrhizal genus *Oidiodendron* and the arbuscular mycorrhizal genus *Ambispora* (Tables S1 and S2).

### Associations of N_2_-fixing bacteria and fungi were more frequent in south-exposed wood blocks

**Figure 4:**
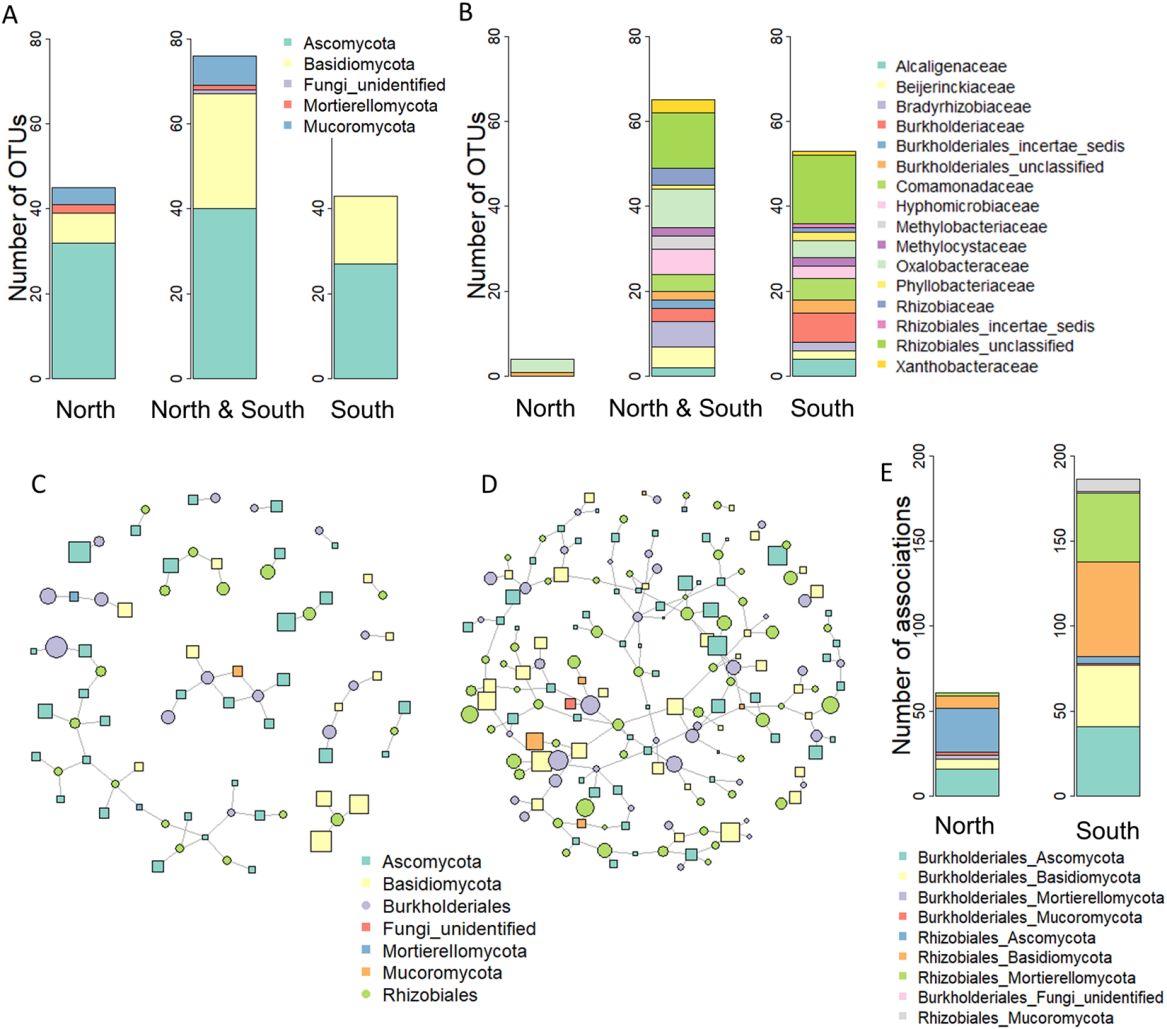
Associations between fungal and N_2_-fixing operational taxonomic units (OTUs) in the *P. abies* experimental wood blocks at the north- and the south-facing site. (**A,B**) Distribution of fungal (**A**) and N_2_-fixing bacterial (**B**) OTUs across the samples collected at north- and south exposure, respectively. The barcharts indicate the taxonomic distribution of the OTUs at the level of phylum (**A**) and family (**B**), respectively. Association networks calculated for the north- (**C**) and the south-facing (**D**) slopes. Every symbol represents a fungal or N_2_-fixing bacterial OTU. Lines indicate the association of two OTUs. Unconnected OTUs were not included into the model. Symbol sizes correspond to the abundances of the OTUs. The binary adjacency matrix of the calculated networks was visualized in R using package igraph^62^ (http://igraph.org). (**E**) The barchart illustrates the taxonomic annotations of the association pairs in the north- and south-exposed wood blocks.

In order to investigate whether the associations of WIF and N_2_-fixing bacteria might have contributed to the differential deadwood decomposition dynamics between the north- and the south-facing site, association networks were calculated. The number of fungal OTUs considered for network calculations was comparable at both slopes (south = 119 OTUs, north = 121 OTUs), as was the number of OTUs that were exclusively detected at each slope (Fig. 4A). A total of 122 N_2_-fixing bacterial OTUs annotated as *Burkholderiales* (49 OTUs) and *Rhizobiales* (73 OTUs) were used for network analyses (Fig. 4B). The richness of N_2_-fixing bacterial OTUs was higher in the south-exposed wood blocks (south = 118 OTUs, north = 69 OTUs). Except for four OTUs, all N_2_-fixing bacterial OTUs detected at the north-facing site were also found at the south-facing one (Fig. 4B).

As we hypothesized, the network of the south-exposed wood samples comprised a higher number of associations between fungal and N_2_-fixing bacterial OTUs (south = 168, north = 61, Table S3). Moreover, a higher percentage of fungal and N_2_-fixing bacterial OTUs were integrated into the south- (75% of all OTUs present) than into the north network (43%). A relatively small number of samples (12 for each slope) were used for network analysis, which might have resulted in a number of relevant associations undetected and some false positive associations between OTUs. This latter aspect is an inherent problem in network analyses^38^. However, these limitations were overcome by applying stringent filtering prior to calculation (Table S4) and using a sparse covariance matrix and strict thresholds in the algorithm. Furthermore, a high number of spurious associations were unlikely to occur considering the coherence of the network across a wide range of strict settings (Table S4). Indeed, we interpreted associations if they were only reoccurring in several differently calculated networks. Moreover, both north and south networks were composed of OTUs with high abundances rather than small OTUs (Table S3).

In addition to size and density, the respective fungal-bacterial associations may also determine the effect on deadwood decomposition. All of the OTU associations were slope-specific (Fig. 4C–E). At both north- and south-facing sites, a comparable number of fungal OTUs were associated to *Burkholderiales* and *Rhizobiales* OTUs (Fig. 4E, Table S3). In the south network 60% of fungal OTUs and 80% of OTUs characteristic for the later stages of decomposition (52 and 104 weeks) had more than one association with a N_2_-fixing bacterial OTU (Table S3). Of those OTUs, 70% of all OTUs and 77% of all indicator OTUs of the later stages showed at least one positive and one negative association in the south network (Table S3). This repetitive pattern emphasizes the ecological relevance of the interactions between fungi and N_2_-fixing bacteria in deadwood and pinpoints that fungi might influence the N_2_-fixing bacterial composition in their habitat.

Basidiomycota, mainly yeasts, that were dominant in terms of abundance at the earlier decomposition stages (Fig. 3A, Table S2) accounted for 41% and 21% of all the associations in the south and north networks, respectively (Fig. 4C–E, Table S3). Wood-decay fungi have been associated with members from the family *Burkholderiaceae*^20^, and co-occurrence between *Burkholderiales* and fungi was also reported for soil environments on a global scale^39^. The increased association to N_2_-fixing bacteria might have provided the basidiomycete fungi with a better access to nitrogen, which is a limited resource in deadwood, especially in the early stages of decomposition^40^, ultimately leading to a faster decay. The Basidiomycota with the highest abundance and strongest associations to *Burkholderiales* in the south network were the yeasts *Guehomyces pullulans* and *Leucosporidiella creatinivora* (= *Rhodotorula creatinivora*) (Table S3). These two species are saprobial generalists degrading plant litter and wood xylan^41^. *L. creatinivora* can degrade phenol and phenol-related compounds in cold habitats^42^, indicating that they could also be capable of degrading lignin. In addition, *L. creatinivora* also had positive associations with *Rhizobiales* (Table S3). These associations were not detected in the north network (Table S3). It is very likely that these associations provide additional competitive advantages for early wood colonization due to the potential N-enrichment of the substrate.

In the later stages of decomposition (52 and 104 weeks), there was an increase in the abundance of Ascomycota at both slopes (Fig. 3A). They were associated with *Burkholderiales* as well as *Rhizobiales* (Fig. 4E). In the south network, a strong positive association was found between the mycorrhizal *Meliniomyces* OTUs and both *Burkholderiales* and *Rhizobiales* (Table S3). These associations were absent in the north network (Table S3). *Meliniomyces* was the taxonomic group with strongest within-group interconnections (Table S3), which might further support the relevance of mycorrhizal fungi in the WIF community (Fig. 3C). Some associations between fungi and *Burkholderiaceae* strains are based on the co-migration of *Burkholderiaceae* strains with fungal hyphae^43,44^. This scenario, in which *Burkholderiaceae* provide protection from antifungal agents while feeding on fungal glycerol, thus supporting fungal growth, might be beneficial for deadwood decomposition.

In contrast thereto, in the north network, characteristic OTUs for the later sampling points with strong associations were *Chlarala - Rhizobiales*, *Pseudogymnoascus - Rhizobiales* and *Mucor* - *Burkholderiales* (Table S3). *Chalara* are anamorphic ascomycetes able to degrade cellulose^45^. However, this group also includes important phytopathogenic fungi, such as *C. fraxinea* causing ash dieback. *Mucor* was often associated with *Burkholderiales* bacteria (Table S3), and this so-called fungus-associated bacteriome is known to be important for the host’s lifestyle and interactions. Shifts in the composition of the fungal-associated bacterial community can slow fungal growth and change fungal secondary-metabolite production^46^.

Generally speaking, the higher number of associations between fungal and N_2_-fixing OTUs likely favoured the ecosystem performance, i.e. deadwood decomposition rate at the south-facing slope when compared to the north-facing one. This is in agreement with an emerging study from Wagg *et al*.^47^, which points towards the importance of microbial inter-kingdom associations as a driver of ecosystem functioning. They demonstrated that microbial networks with a greater number of associations between bacterial and fungal taxa contributed more to support multiple ecosystem functions simultaneously related to soil nutrient cycling than simpler or low-diversity networks.

## Conclusions

In conclusion, our findings suggest that the enhanced decomposition of *P. abies* deadwood at the south-facing slope was related to a higher abundance of wood-degrading OTUs such as *Mycena*, along with a higher prevalence of mycorrhizal and root endophytic OTUs at the later decomposition stages within the two-year period. Although our observations only covered one site per slope and cannot be extrapolated to a landscape scale, they provide evidence that the colder conditions at the northern slope might have delayed the succession, and thus the establishment of an efficient wood-degrading fungal community. Moreover, the higher abundance of *Mucor* spp. and *Trichoderma* on the north-exposed wood blocks further supported the taxonomic and functional differences between the two study sites. Additionally, a higher number of associations between WIF and N_2_-fixing OTUs was found in the south network. This could have provided additional competitive advantages, especially for early wood colonization and decomposition at the south-facing site.

## Material and Methods

### Study area

The investigation area is located in the southern Alpine belt in northern Italy (Val di Rabbi, Trentino). The two selected sites were located at similar altitudes at north- (1,930 m a.s.l, 46°22.756′) and south-facing (1,995 m a.s.l., 46°21.321′) slopes. They are part of an observation network with a comprehensive characterization of the soils^25,48^, classified as Episkeletic Podzol and Skeletic Umbrisol. Table 2 gives an overview of the main characteristics of the study sites.

**Table 2:** Characteristics of the two study sites at the north and the south facing slopes in Val di Rabbi^90^.

Sites	Altitude (m a.s.l.)	Aspect (°N)	Slope (°)	MAP (mm y^−1^)	MAAT (° C)	MAST (° C)	Parent material	Dominating tree species	Land use	Soil classification
North exposure	1930	20	12	1180	1.4	5.0	Paragneiss debris, Moraine material	*Larix decidua*	Originally used as pasture	Episkeletic Podzol
South exposure	1995	160	25	1180	3.4	6.4	Paragneiss debris	*Larix decidua*	Ex-pasture, natural forest (ecological forestry)	Skeletic Umbrisol

MAP = mean annual precipitation; MAAT = mean annual air temperature; MAST = mean annual soil temperature.

### Mesocosm experimental set-up

From a climosequence experiment with a soil mesocosms set up from August 2012 we chose two sites^24,28^. Mesocosm experiments have already been used in terrestrial environments owing to their potential to mimic aspects of a real-world environment under controlled conditions and by allowing inferential testing of selected factors^49^. However, small-scale mesocosm experiments may result in a reduced ecological complexity relative to the “reference” ecosystem, which limits their potential to address and extrapolate findings to larger spatial and temporal scales^50^.

The mesocosm tubes (Ø 10.2 cm, 25.0 cm long PVC tubes) were inserted into the soil, >1 m from large trees and >0.5 m from adjacent tubes, leaving a rim of about 1 cm at the surface (Figs. 1, S1). The installed soil mesocosms were allowed to equilibrate for one year. Prior to starting the mesocosm trial (June 2013), equally sized (5 × 5 × 2 cm) Norway spruce (*Picea abies* (L.) Karst) wood blocks were cut from one log of one single forest tree grown in the study area. Until their placement on the top of the soil mesocosms, the wood blocks were stored at room temperature. Three wood blocks (t0) were stored at −20 °C for molecular analysis. All the wood blocks showed similar physico-chemical characteristics at t0 as reported by Probst *et al*.^28^. At each study site and prior to the placement of the wood blocks into the mesocosms, three composite soil samples (made out of five sub-samples each) were randomly taken in the experimental area (at a distance between 0.25 m and 0.5 m from the mesocosm tubes) and referred as to t0. The mesocosms, including the wood blocks and the uppermost topsoil layer (0–5 cm), were destructively sampled (three field replicate mesocosms at each time point) after 12 (t1), 25 (t2), 52 (t3) and 104 (t4) weeks at both the north- and the south-facing sites (Fig. 1). All the samples (wood blocks and soil) were placed into polyethylene bags and transported on ice to the laboratory. The wood blocks were air-dried at room temperature in an incubator prior to cut-milling (4 mm; Pulverisette, Fritsch), and the soil samples were sieved (<2 mm). All the samples were stored at −20 °C until analyses. The physico-chemical characterization of wood and soil samples was done as reported in Probst *et al*.^28^. Moreover, the soil temperature was measured close to the mesocosms between June 2013 and July 2014 in 3 h intervals with temperature loggers, iButton as shown in Schmid *et al*.^51^, placed 10 cm below the soil surface.

### DNA extraction

Total DNA was extracted from three field replicates of soil (0.2 g) and wood samples (0.1 g) by using commercial kits (MP Biomedicals), FastDNA Spin Kit for soil and FastDNA Kit for wood, respectively, in combination with the FastPrep Instrument as described by Ascher *et al*.^52^ For wood samples, one 1/4 ceramic sphere (MP Biomedicals cat # 6540–424) was added to the lysing tubes to guarantee an accurate disruption of the woody tissue and the break-up of microbial cells. All DNA extracts (wood and soil) were purified using the GeneClean procedure (FastDNA Spin Kit for soil) and analysed in terms of quality and quantity as reported by Bardelli *et al*.^25^.

### Illumina MiSeq sequencing and bioinformatics pipeline

Fungal communities were analysed by the amplification and sequencing of the internal transcribed spacer 2 (ITS2) region using the ITS3 and ITS4 primer set^53^ under the following cycling conditions: an initial denaturation at 95 °C for 3 min, followed by a two-step PCR procedure of 35 cycles of denaturation at 98 °C for 20 s, primer annealing at 56 °C for 30 s, and extension at 72 °C for 30 s with a final elongation step at 72 °C for 5 min. PCR products were then purified, quantified and pooled in equimolar concentrations for sequencing on an Illumina MiSeq using the 2 × 250 bp paired-end approach (Microsynth AG, Switzerland).

Raw Illumina MiSeq paired fungal sequences were demultiplexed and then merged using the command “fastq_mergepairs”, which is implemented in USEARCH pipeline^54^. Afterwards, fungal sequences were quality-filtered and clustered into operational taxonomical units (OTUs) using USEARCH pipeline and UPARSE algorithm^54^. Briefly, quality filtering was carried out by trimming sequences to 300 bp and allowing a maximum e-value of 0.5. Filtered sequences were then dereplicated and sorted by abundance. Singletons were removed prior to OTU determination at 97% sequence identity. Chimeric representative sequences from the OTUs were removed using UCHIME^55^. Finally, original sequences were mapped to OTUs at the 97% identity threshold to obtain one OTU table. The taxonomic affiliation of each fungal OTU was obtained using Ribosomal Database Project (RDP) taxonomic classifier^56^ against UNITE Fungal ITS train set 07-04-2014 using a confidence threshold of 50%. All OTUs were classified as fungi and were thus retained. The sequence data were deposited in the GenBank SRA database under accession number PRJNA427454.

FUNGuild was used to taxonomically parse fungal OTUs by ecological guild^32^. A frequency table was created containing the number of OTUs assigned to each guild for each of the studied samples. In addition, the abundance of OTUs assigned to the different guilds was summed up for each sample in order to ensure that both abundance and frequency follow the same pattern. In addition to the guild annotations obtained from FUNGuild, we manually summarized guilds that were ecologically comparable and more informative for deadwood samples. For example, OTUs categorized as “Dung saprotroph; undefined saprotroph; wood saprotroph”, “Dung saprotroph; wood saprotroph”, “Ectomycorrhizal; wood saprotroph” and “Wood saprotroph” were summarized as “Wood saprotroph”. As a result, the functionally annotated OTUs were summarized into nine categories: Endophyte, Lichenized, Mycorrhiza, Pathogen, Saprotroph, Pathogen-Saprotroph, Wood-Saprotroph, Wood-Saprotroph-Pathogen and others. A complete overview can be found in Table S1. Using both sets of categories we compared the sample groups according to the relative read abundances of OTUs and the number of OTUs in each category.

### Statistical analyses

The bioinformatics analysis resulted in an OTU table listing the read abundance of all the detected OTUs in all of the samples. The sequencing depth of the individual samples varied between a minimum of 44,165 and a maximum of 158,573 paired reads. The average sequencing depth over all the samples was 92,149 paired reads. For each sample, OTUs > 5 reads were considered. One soil sample (25 weeks’ incubation, south exposure, replicate 2) was not considered for further analyses. A very high number of OTUs occurring exclusively in this sample suggested some kind of random contamination. The sequencing depth of the wood samples did not differ between study sites (p = 0.372) or time points (p = 0.181). OTUs which had zero abundance after filtering were removed from the dataset. Based on the filtered OTU table, the fungal richness (number of OTUs) and Shannon diversity were calculated for each sample using the R package vegan^57^. A metadata set was available containing the environmental variables including pH, moisture and cellulose content for the studied samples^28^. Data were imported into R 3.6 according to R Core Team^58^ for statistical analysis referring to fungal community data. Basic linear models were calculated in order to assess the relation of the experimental factors (slope exposure and time) with regard to the environmental variables, and fungal richness and Shannon diversity. Basic linear modelling was also used to assess the influence of environmental variables on both fungal richness and Shannon diversity. In all models, experimental factors were applied as fixed factors. The interaction effect of the abovementioned experimental factors was also considered within the framework of the present study. For single variables, such as fungal richness and Shannon diversity, sample groups were compared using non-parametric Wilcoxon (exposure) or Kruskal-Wallis test (time). The multidimensional character of both taxonomic and functional fungal community composition of the wood and soil samples was projected into two dimensions using non-metric multidimensional scaling (NMDS). A matrix was generated containing the Bray-Curtis dissimilarities between samples based on OTU abundances and functional frequencies (OTUs per functional category). Based on this matrix, the NMDS was calculated using vegan’s metaMDS function^57^. The amount of variance explainable by the experimental factors (slope exposure and time) and environmental variables was calculated using permutational multivariate analysis of variance (adonis) on Bray-Curtis distances^57^.

Fungal taxonomic groups and single OTUs characteristic for the wood blocks at t0, early (12 and 25 weeks) and late (52 and 104 weeks) decomposition stages at the north- and the south-facing site were identified using the linear discriminant analysis (LDA) effect size (LEfSe)^59^ online tool (http://huttenhower.sph.harvard.edu/galaxy/). For LEfSe results, a confidence interval of 99.5% was applied.

Network analyses were performed to assess the potential associations between fungal and N_2_-fixing bacterial OTUs in the *P. abies* wood blocks. Bacterial OTUs were selected from the dataset from Probst *et al*.^28^. Briefly, bacterial V3/4 16 S sequences were summarized to 97% similar OTUs. The dataset was filtered for N_2_-fixing bacterial OTUs by their taxonomic annotation to either *Burkholderiales* or *Rhizobiales* on the level of order according to RDP reference database applying a cut-off of 80. Association networks were calculated for each slope based on these OTU tables applying R package SpiecEasi^60,61^. As the sample number was relatively small for network analysis, we calculated association networks across a wide range of filter criteria and thresholds (Table S4). Both OTU tables were filtered for OTUs with minimum abundances of 2, 3, 5 and 10 reads in at least 2, 3 and 5 samples prior to network calculations. The networks were fitted applying neighbourhood selection (MB method) and the best model was selected using Stability Approach to Regularization Selection (StARS). Thresholds for lambda were 20 (default), 30, 40 and 50. Lambda minimum log ratios of 0.01, 0.05 (default) and 0.001 were tested. The coherence of the north and south networks was observed following the number of edges and nodes as well as the density of the networks (Table S4). The final network was chosen as representative across thresholds with the highest congruency to standard protocols and appropriate for the data. Consequently, an OTU abundance > 5 reads present in at least 3 samples, which is at least all biological replicates per time point, was applied. Lambda of 40 was chosen based on the SpiecEasi tutorial on gut microbiome. The log ratio was reduced to 0.01 in order to apply very strict criteria. OTU pairs and the directionality of their association were extracted from the adjacency matrix (symBeta(getOptBeta)) of each network. The binary adjacency matrix was subjected to igraph^62^ for visualization and further analysis, such as the diameter and modularity of the graph.

## Supplementary information

Supplementary information.

Supplementary Table 1

Supplementary Table 2

Supplementary Table 3

Supplementary Table 4
