# Publisher Correction: Fungal communities and their association with nitrogen-fixing bacteria affect early decomposition of Norway spruce deadwood

**DOI:** 10.1038/s41598-020-66769-1

**Published:** 2020-06-16

**Authors:** María Gómez-Brandón, Maraike Probst, José A. Siles, Ursula Peintner, Tommaso Bardelli, Markus Egli, Heribert Insam, Judith Ascher-Jenull

**Affiliations:** 1 Grupo de Ecoloxía Animal (GEA), Universidade de Vigo, E-36310 Vigo, Spain; 2 Department of Microbiology, University of Innsbruck, Technikerstraβe 25, A-6020 Innsbruck, Austria; 3 Department of Plant and Microbial Biology, University of California at Berkeley, Berkeley, CA 94720 USA; 4 Dipartimento di Scienze e Tecnologie Agrarie, Alimentari, Ambientali e Forestali (DAGRI), University of Florence, Piazzale delle Cascine 18, I-50144 Florence, Italy; 5 Council for Research and Experimentation in Agriculture (CREA-ZA), Via A. Lombardo 11, I-26900 Lodi, Italy; 6 Department of Geography, University of Zürich, Winterthurerstraße 190, CH-8057 Zürich, Switzerland

Correction to: *Scientific Reports* 10.1038/s41598-020-64808-5, published online 15 May 2020

In the original version of this Article, Maraike Probst was incorrectly listed as the corresponding author. The correct corresponding author for this Article is María Gómez-Brandón. Correspondence and request for materials should be addressed to mariagomez@uvigo.es.

In addition, four Supplementary Tables were omitted from the original version of this Article. These tables contain results from FUNGuild analysis, results from Linear discriminant analysis, a list of fungal – N2-fixing-bacterial OTUs associated to each other in the SpiecEasi network analysis, and an overview of the north and south network respectively.

This has been corrected in the HTML and PDF versions of the Article.

