# Supplementary material for: Fungal communities and their association with nitrogen-fixing bacteria affect early decomposition of Norway spruce deadwood
Source: Sci Rep. 2020 May 15;10:8025. doi: 10.1038/s41598-020-64808-5 (PMC7228967; doi:10.1038/s41598-020-64808-5)
Supplement: Supplementary file 1 — Supplementary information. [file 41598_2020_64808_MOESM1_ESM.docx]

Supplementary Information

**Fungal communities and their association with nitrogen-fixing bacteria affect early decomposition of Norway spruce deadwood**

**María Gómez-Brandón^1,2†*^, Maraike Probst^2†^, José A. Siles^3^, Ursula Peintner^2^, Tommaso Bardelli^2,4,5^, Markus Egli^6^, Heribert Insam^2^ & Judith Ascher-Jenull^2^**

^1^Grupo de Ecoloxía Animal (GEA), Universidade de Vigo, E-36310, Vigo, Spain

^2^Department of Microbiology, University of Innsbruck, Technikerstraβe 25, A-6020 Innsbruck, Austria

^3^Department of Plant and Microbial Biology, University of California at Berkeley, Berkeley, CA 94720, USA

^4^Dipartimento di Scienze e Tecnologie Agrarie, Alimentari, Ambientali e Forestali (DAGRI), University of Florence, Piazzale delle Cascine 18, I-50144 Florence, Italy

^5^Council for Research and Experimentation in Agriculture (CREA-ZA), Via A. Lombardo 11, I-26900 Lodi, Italy

^6^Department of Geography, University of Zürich, Winterthurerstraße 190, CH-8057 Zürich, Switzerland

†These authors contributed equally to this paper

Correspondence: Dr. María Gómez Brandón, Grupo de Ecoloxía Animal (GEA), Universidade de Vigo, E-36310, Vigo, Spain.

*E-mail address*: mariagomez@uvigo.es (M. Gómez-Brandón)

#
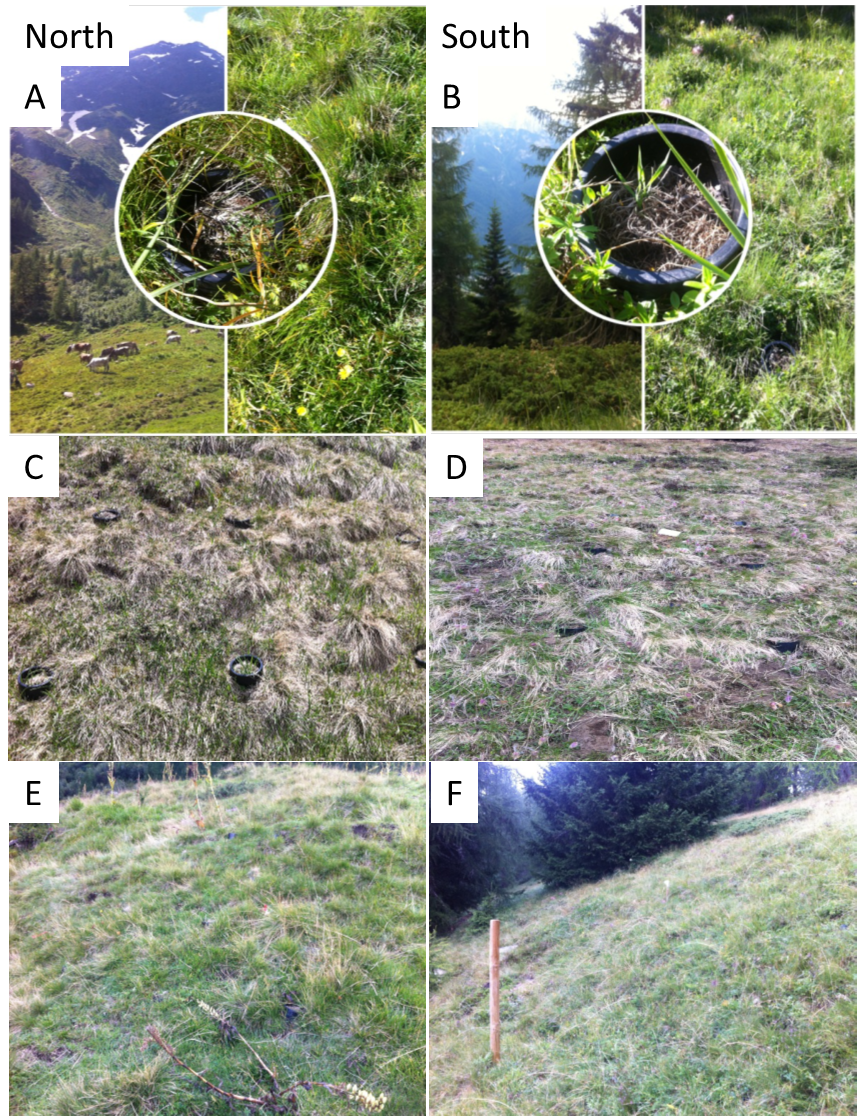


**Fig. S1** Overview of the north- and south-facing site at the different time points. (A, B) June 2013: Sampling sites at t0, start of the mesocosm experiment. (C, D) August 2013: Sampling sites after 12 weeks of the wood blocks´ placement. (E, F) July 2014: Sampling sites after 52 weeks of the wood blocks´ placement.

#
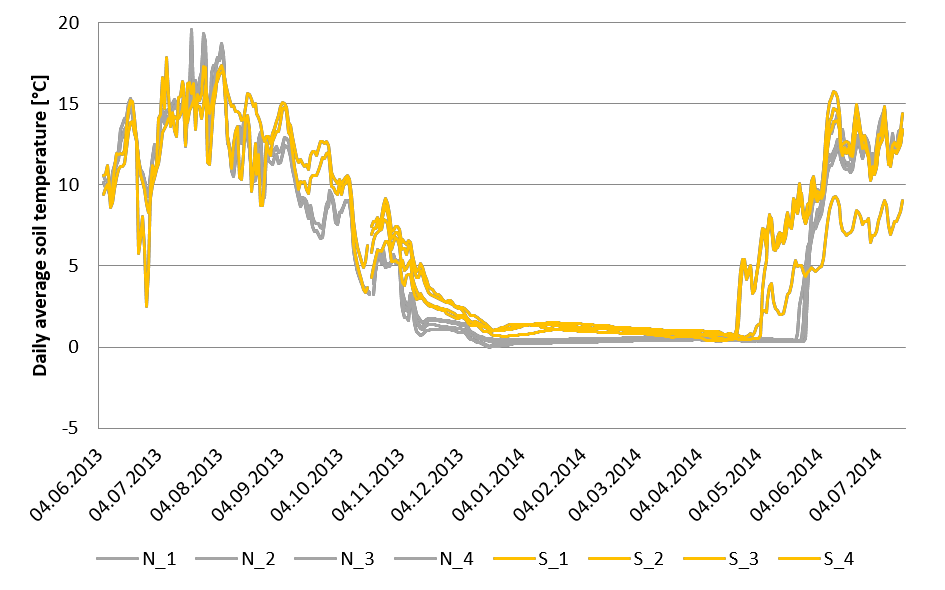


**Fig. S2** Overview of the soil temperature profile at the north (N)- and the south (S)-facing site. The temperature measurements were taken between June 2013 and July 2014, which can be considered as a representative year of the observational period of the mesocosm monitoring trial.

#
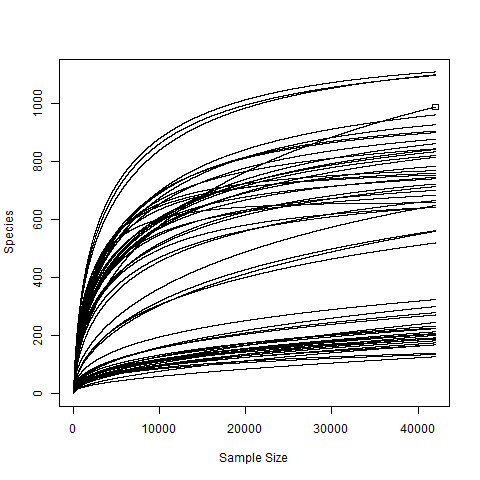


**Fig. S3** Rarefaction curves for the *P. abies* experimental wood blocks and the underlying soil samples (0-5 cm). These curves indicate that the sequencing depth was sufficient for all of the samples in the full dataset

# Functional annotation

The functional role of wood inhabiting fungi (WIF) was predicted using FUNGuild, which assigns functional, ecological roles based on the taxonomic annotation of the Illumina-generated ITS reads. For further analysis, we used the guild annotation output of FUNGuild. In order to get a more mechanistic insight into the differences in deadwood decomposition between the north- and the south-facing site, we summarized FUNGuild categories that were ecologically comparable in our deadwood environment (Fig. S4A,B).

By calculating NMDS analysis using the number of OTUs within the FUNGuilds as variables (Fig. S4C), we recreated the grouping observed in the NMDS plot based on OTUs (Fig. 3B). Based on Bray-Curtis distances, 18% of the variance could be attributed to the factor time (p_Adonis_ = 0.001). The differences in terms of slope exposure accounted for 10% (p_Adonis_ = 0.005). The interaction effect of time and exposure explained an additional 10% of the overall variance (p_Adonis_ = 0.013). In agreement with the WIF composition based on OTUs, only variances in pH correlated with functional variations between wood blocks´samples (R^2^ = 0.126, p_Adonis_ = 0.01). Repeating the analysis on OTU abundances per functional category, rather than the number of OTUs per functional category, also resulted in a clear separation of WIF functional compositions over time (R^2^ = 0.29, p_Adonis_ = 0.0001). However, the exposure effect was absent (R^2^ = 0.067, p_Adonis_ = 0.059). It was likely that 30% of OTUs being annotated were sufficient to resolve this pattern. However, the fact that the OTU frequency in 40 functional categories provided the same pattern as observed using 3,555 OTUs underlined that there were substantial differences in the deadwood decomposition process and the WIF community involved when compared the north- and the south-facing site with regard to the same period of time.

Saprotrophs was the biggest functional group in terms of read abundance and OTU frequency in all of the wood blocks´ samples (Fig. S4A,B). Almost half of the all indicator species identified using Linear discriminant analysis of size effect (LefSe) were functionally annotated (46%, Tables S1 and S2). The majority of them were classified as saprotrophic (75%). The most important indicator species at the earlier time points were saprotrophic basidiomycetes *Sistotrema, Trametes* and (less abundant) *Heterobasidion* OTUs. Functional annotation can sometimes be misleading, as many fungal taxa are mixotroph, having multiple functions. *Sistotrema,* for example, can also be ectomycorrhizal and *Heterobasidion* is also known as an aggressive tree pathogen (Table S1). Other genera like *Epicoccum* and *Embellisia* increased in abundance in the north-facing site when compared to the south-facing one (Tables S1 and S2). These potential pathogenic genera have an important role as mixotrophic saprotrophs: *Epicoccum* has been reported as psychrotolerant saprotroph on litter under snow cover, and has been attributed an important role in C cycle during winter^1^. *Embellisia* has good lignin degrading capabilities as it is a good degrader of polychlorinated aromatic compounds^2^.


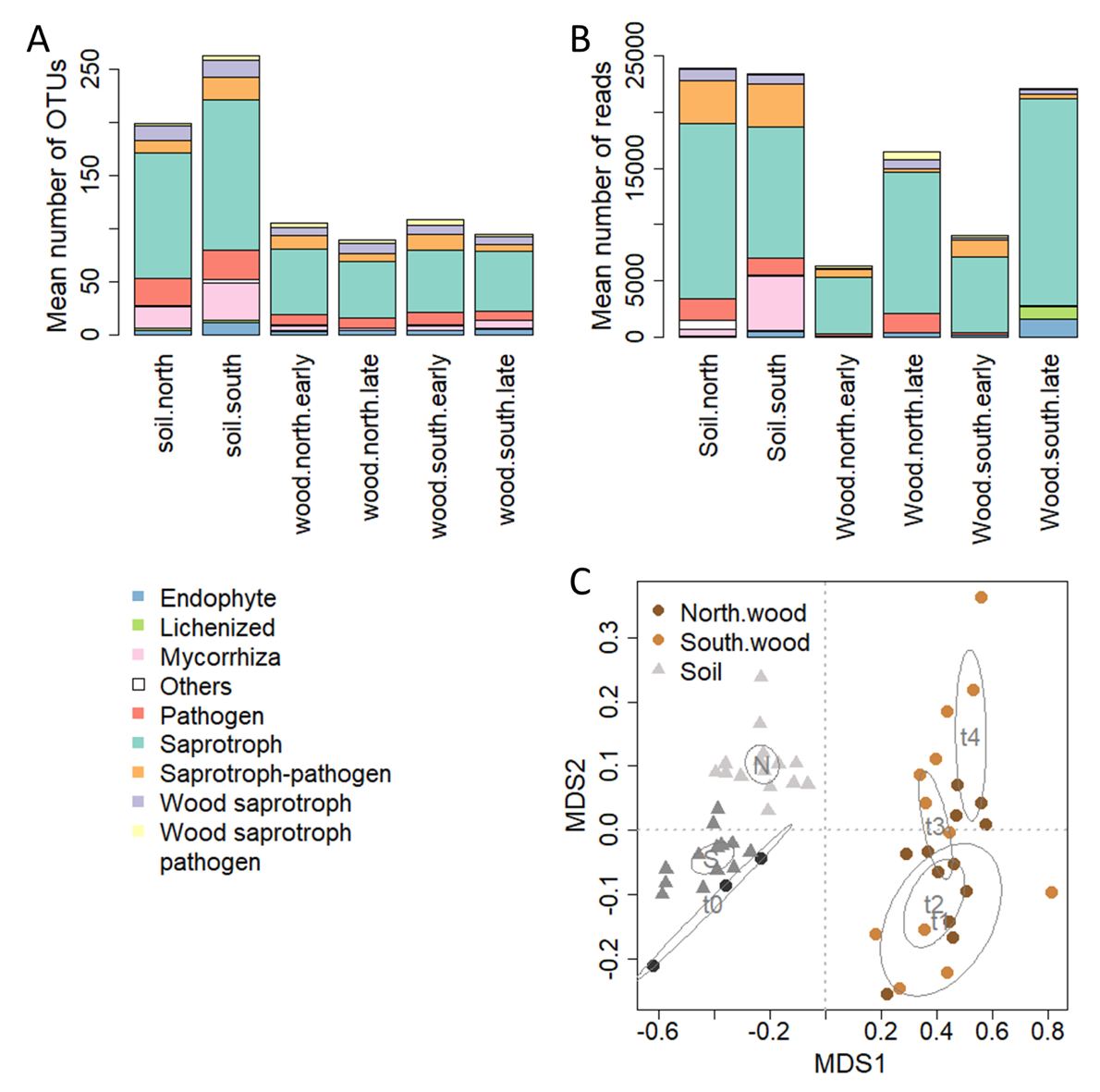


**Fig. S4.** Functional composition of the *P. abies* experimental *w*ood blocks and the underlying soil samples. (A, B) Overview of the functional composition of all sample groups. The functional composition of the samples was illustrated as the frequency (A) and abundance (B) of OTUs assigned to the guilds. (C) NMDS based on Bray-Curtis dissimilarities between the functional compositions of the samples based on OTU frequencies in the guilds. The lowest stress was 0.083. The iteration converged after 20 tries. For NMDS, original FUNGuild annotations were used as variables. For barcharts, FUNGuild categories were manually summarized into fewer categories in order to simplify visualization and data accession (Table S1).

**References**

1. Uchida, M., Mo, W., Nakatsubo, T., Tsuchiya, Y., Horikoshi, T., & Koizumi, H. Microbial activity and litter decomposition under snow cover in a cool-temperate broad-leaved deciduous forest. *Agr Forest Meteorol* **134** (1-4),102-109 (2005).

2. Garon, D., Krivobok, S., & Seigle-Murandi, F. Fungal degradation of fluorene. *Chemosphere* **40** (1), 91-97 (2000).
